# Rhabdomyosarcoma in Children: A Retrospective Analysis of 12 Cases

**DOI:** 10.7759/cureus.112761

**Published:** 2026-07-16

**Authors:** Latifa Miraoui, Anass Haloui, Maria Rkain, Amal Bennani

**Affiliations:** 1 Department of Anatomopathology, Mohammed VI University Hospital, Faculty of Medicine and Pharmacy, Mohammed I University, Oujda, MAR; 2 Department of Pediatrics, Mohammed VI University Hospital, Faculty of Medicine and Pharmacy, Mohammed I University, Oujda, MAR

**Keywords:** anatomical pathology, diagnostic, epidemiology, prognosis, rhabdomyosarcomas

## Abstract

Objective

This study aimed to describe the clinicopathological characteristics, immunohistochemical profile, treatment, and outcomes of 12 pediatric rhabdomyosarcoma (RMS) cases diagnosed at our institution and to compare our findings with those reported in the literature.

Materials and methods

The study involved a retrospective analysis of 12 cases of histologically confirmed RMS diagnosed between November 2018 and September 2025 at the Department of Anatomical Pathology at the Mohammed VI University Hospital Center in Oujda.

Results

The sex ratio was 1:1, and the median age at diagnosis was two years and six months. Children aged between one and five years represented eight cases (66.6%). Tumor size exceeded 5 cm in eight patients(66.6%). The most common anatomical location was the head and neck (non-parameningeal) region, accounting for three cases (25%), followed by the orbit, extremities, and bladder, with two cases each (16.7%). The genitourinary tract (excluding the bladder and prostate), abdomen, and trunk each represented one case (8.3%). Embryonal RMS was the predominant histological subtype, accounting for eight cases (66.6%), whereas alveolar RMS and the botryoid variant of embryonal RMS each represented two cases (16.7%). Risk stratification revealed an equal distribution among the four categories, each accounting for three cases (25%): low-risk subset 1, low-risk subset 2, intermediate risk, and high risk. All patients received chemotherapy, whereas five (41.7%) also underwent surgery, and six (50%) received radiotherapy.

Conclusions

RMS is the third most common pediatric tumor after neuroblastoma and nephroblastoma. Histopathological and immunohistochemical examinations remain essential for accurate diagnosis, tumor classification, and therapeutic decisions. Treatment relies on a multidisciplinary approach combining chemotherapy with surgery and/or radiotherapy based on risk stratification and other prognostic factors. Despite advances in management, RMS remains one of the most common pediatric soft tissue sarcomas and continues to represent a significant therapeutic challenge.

## Introduction

Rhabdomyosarcoma (RMS) is a malignant mesenchymal tumor characterized by skeletal muscle differentiation. It accounts for approximately 7% of all childhood solid malignancies and is the third most common pediatric malignancy after neuroblastoma and nephroblastoma. RMS is also the most common pediatric soft tissue sarcoma, accounting for more than 50% of all soft tissue sarcomas in children [1,2]. The annual incidence of RMS is estimated at 4.3 cases per million children, with approximately 70% of cases occurring before the age of 10 years [1,3]. Although it can arise at virtually any anatomical site, the head and neck region, genitourinary tract, and extremities are among the most commonly affected locations [4]. The etiology of RMS remains largely unknown; however, genetic susceptibility and molecular alterations have been implicated in its pathogenesis [5].

Advances in histopathology, immunohistochemistry, and molecular genetics have considerably improved the diagnosis and classification of RMS. Current treatment strategies rely on a multidisciplinary approach combining chemotherapy, surgery, and radiotherapy, guided by risk stratification systems that integrate clinical, pathological, and molecular characteristics [6]. Prognosis depends on several factors, including histological subtype, metastatic status at diagnosis, patient age, tumor location, tumor size, and resectability [1]. The present study aims to describe the epidemiological, histopathological, clinical, and therapeutic characteristics of RMS diagnosed at the Department of Anatomical Pathology at the Mohammed VI University Hospital Center in Oujda and to compare our findings with those reported in the literature.

## Materials and methods

This retrospective descriptive study was performed in the Department of Anatomical Pathology of Mohammed VI University Hospital Center in Oujda over a period of 6 years and 10 months, spanning from November 2018 to September 2025. The study included all patients younger than 15 years with a histologically confirmed diagnosis of rhabdomyosarcoma (RMS) made by the Department of Anatomical Pathology during the study period. A total of 12 cases fulfilled the inclusion criteria. Patients aged 15 years or older, those with tumors other than RMS, and cases without histological confirmation were excluded.

Clinical, radiological, pathological, therapeutic, and follow-up data were retrospectively retrieved from patients' medical records, pathology reports, surgical logs, and imaging archives. The variables extracted included age, sex, clinical presentation, primary tumor site, tumor size, radiological features, TNM stage, histological subtype, regional lymph node status, distant metastases, treatment regimens, recurrence, follow-up duration, and survival outcomes.

All biopsy and surgical specimens were fixed in 10% neutral-buffered formalin, routinely processed, embedded in paraffin, sectioned at 3-4 μm, and stained with hematoxylin and eosin (H&E). Histological diagnoses were rendered according to the World Health Organization (WHO) Classification of Soft Tissue and Bone Tumors. Immunohistochemical analysis was performed on formalin-fixed paraffin-embedded tissue sections using an antibody panel selected according to the morphological differential diagnosis, including desmin and myogenin, with additional markers applied when needed.

Molecular testing was not performed routinely, as these techniques were unavailable at our institution during the study period, owing to financial and technical constraints. Consequently, diagnoses were based on histomorphological and immunohistochemical findings alone. Patients were stratified according to the Children's Oncology Group (COG) risk classification system, which incorporates primary tumor site, TNM stage, postoperative clinical group, histological subtype, tumor size, regional lymph node involvement, and the presence of distant metastases.

## Results

Twelve pediatric patients with histologically confirmed RMS were included in this study. The epidemiological, clinical, histopathological, and therapeutic characteristics of these cases were analyzed. The main findings regarding patient demographics, tumor features, histological subtypes, immunohistochemical profiles, risk stratification, treatment modalities, and outcomes are presented below. The median age at diagnosis was two years and six months (range: six months to 14 years and four months). Children aged between one and five years accounted for eight cases (66.6%). The sex ratio was 1:1, indicating an equal distribution between males and females.

**Figure 1:**
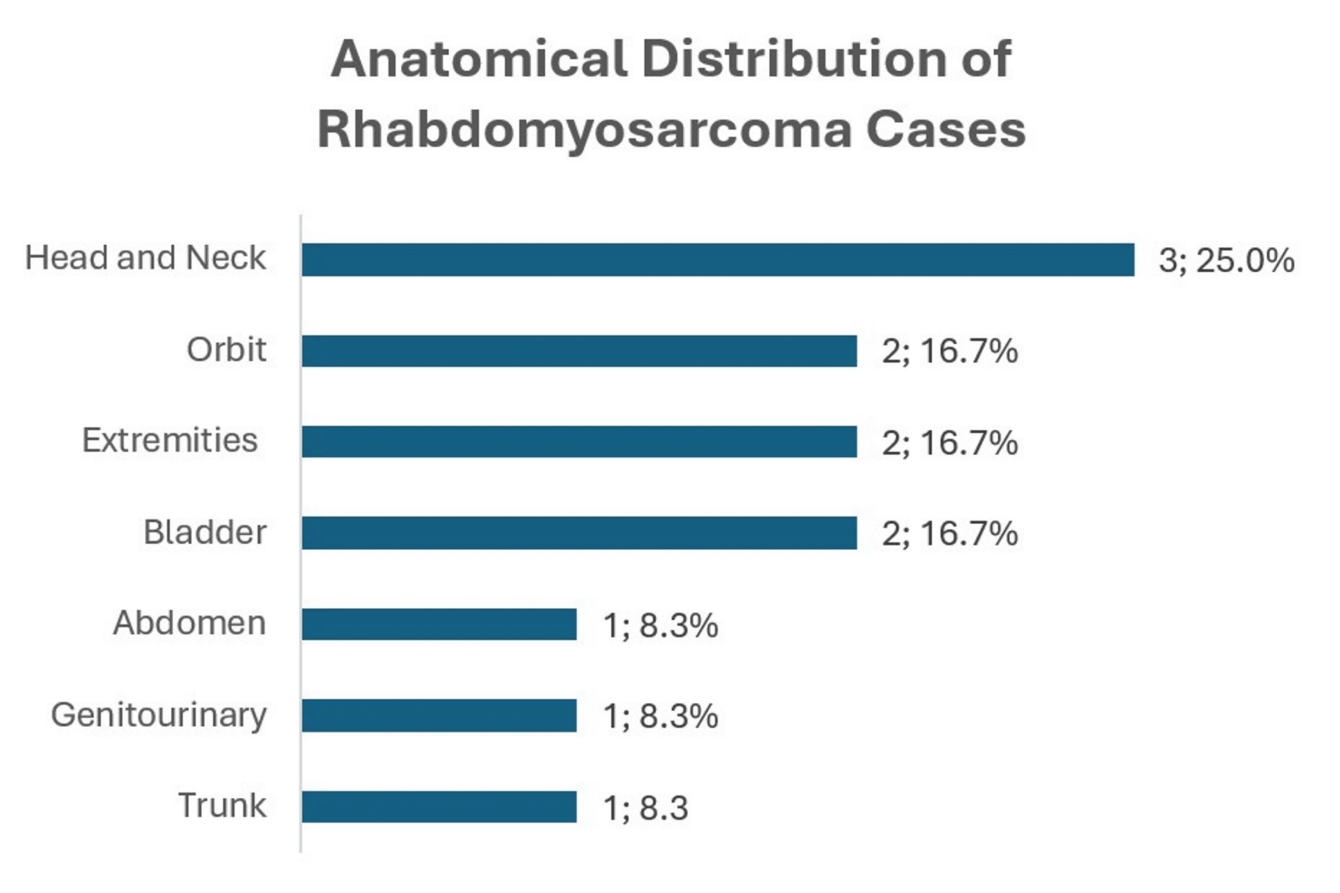
Anatomical distribution of rhabdomyosarcoma cases (n = 12)

Tumor size exceeded 5 cm in eight patients (66.6%), while four patients (33.3%) presented with tumors measuring less than 5 cm. Regarding anatomical distribution, the head and neck (non-parameningeal) region was the most frequently affected site, accounting for three cases (25%). The orbit, extremities, and bladder each represented two cases (16.7%), whereas the genitourinary tract (excluding the bladder and prostate), abdomen, and trunk each accounted for one case (8.3%), as shown in Figure 1. At the time of diagnosis, metastatic disease was present in seven patients (58.3%).

**Figure 2:**
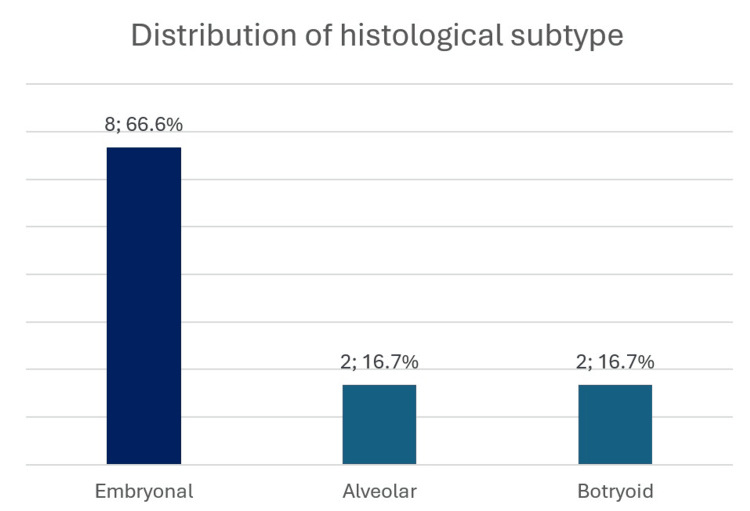
Distribution of histological subtypes among rhabdomyosarcoma cases (n = 12)

**Figure 3:**
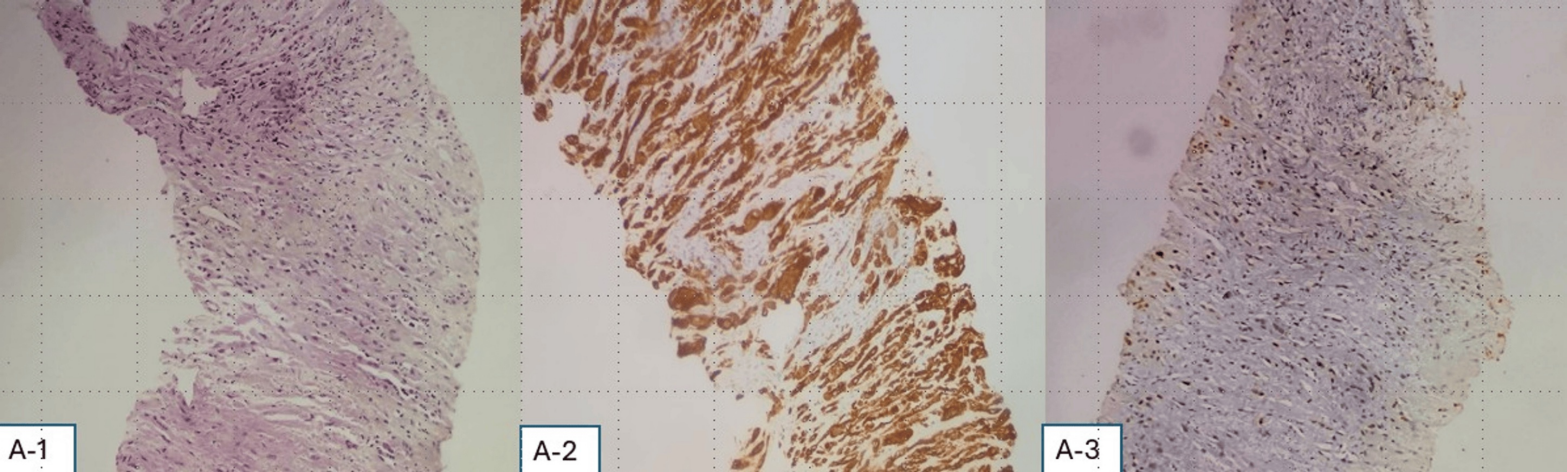
Embryonal subtype A-1: H&E x20; A-2: desmin x20; A-3: myogenin x20 H&E: hematoxylin and eosin

**Figure 4:**
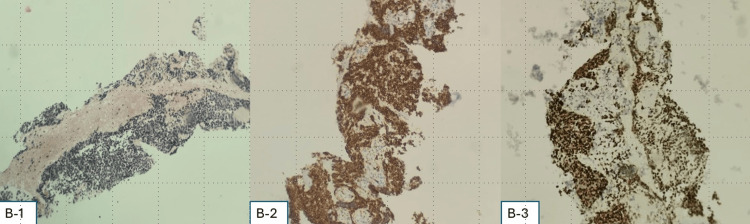
Alveolar subtype B-1: H&E x20; B-2: desmin x20; B-3: myogenin x20 H&E: hematoxylin and eosin

**Figure 5:**
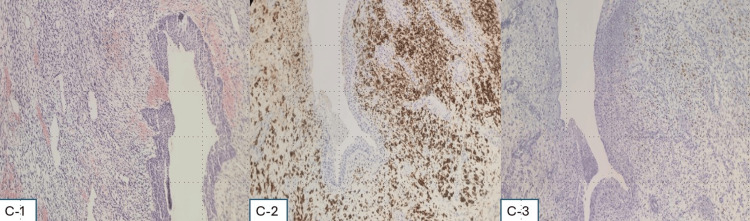
Botryoid variant C-1: H&E x20; C-2: desmin x20; C-3: myogenin H&E: hematoxylin and eosin

As shown in Figure 2, the embryonal subtype was the predominant histological variant (Figure 3), accounting for eight cases (66.6%). It was primarily observed in the head and neck (non-parameningeal) region. The alveolar subtype (Figure 4) represented two cases (16.7%) and was exclusively located in the extremities and trunk. The botryoid variant of embryonal RMS (Figure 5) also accounted for two cases (16.7%) and was confined to the bladder and genitourinary tract.

Immunohistochemical analysis (Figures 3-5) demonstrated diffuse positivity for desmin and Myogenin in all twelve cases (100%). Risk stratification revealed an equal distribution across the four risk categories. Low-risk subset 1, low-risk subset 2, intermediate-risk, and high-risk patients each accounted for three cases (25%) of the study population.

All patients received chemotherapy as part of their treatment regimen, as shown in Table 1. Surgical intervention was performed in five cases (41.7%), while six patients (50%) received radiotherapy. Chemotherapy-related adverse events included isolated neutropenia in six patients (50%), severe anemia in four (33.3%), febrile neutropenia in two cases (16.7%), and interstitial lung disease in two cases (16.7%). Overall, four deaths were recorded, representing 33.3% of the study population.

**Table 1:** Treatment modalities administered to patients with rhabdomyosarcoma (n = 12)

Treatment modality	N (%)
Chemotherapy	12 (100.0)
Radiotherapy	6 (50.0)
Surgery	5 (41.7)

## Discussion

In the current WHO classification, four histological types of RMS are recognized and classified as follows: embryonal, alveolar, pleomorphic, and spindle cell/sclerosing RMS. Embryonal RMS is the most common subtype, characterized by the absence of recurrent fusion genes, diffuse desmin expression, and focal positivity for myogenin and myoblast determination protein 1 (MyoD1) [7,8]. It also shows recurrent chromosomal gains (2, 7, 8, 11, 12, 13, and 20) in 25-50% of cases and losses (9, 10) in some cases [9]. Alveolar RMS, in contrast, is a fusion-positive sarcoma that displays characteristic PAX3-FOXO1 (56-85%) or PAX7-FOXO1 (6-10%) fusions [9,10] and demonstrates strong and diffuse nuclear staining for myogenin and MyoD1 [9,11].

Pleomorphic RMS is uncommon in pediatric patients, shows marked cellular pleomorphism [7,12], and is genetically characterized by a poor prognosis due to its complex karyotype with copy number alterations and unbalanced structural alterations [9,13]. The spindle cell/sclerosing subtype, also uncommon in children, is characterized by ribbon-shaped cells or fascicular/herringbone patterns [7,12], exhibits distinctive spindle-shaped morphology, and includes a recently described variant exhibiting EWSR1/FUS-TFCP2 fusions and a predilection for intraosseous locations [9,14].

From an epidemiological perspective, nearly two-thirds of RMS cases are diagnosed in children aged six years or younger, with a lower peak incidence in early to mid-adolescence. The disease is slightly more common in boys (11.8 per million) than in girls (10.3 per million), with reported sex ratios ranging from 1.4 to 1.7 [1,3]. In the present study, the median age at diagnosis was two years and six months (range: six months to 14 years and four months), and 66.6% of patients were between one and five years of age. These results are consistent with those reported by the Intergroup Rhabdomyosarcoma Study (IRS III-IV), in which more than two-thirds of patients were diagnosed before the age of six. In contrast, in our cohort, males and females were equally represented (sex ratio = 1:1). However, given the small sample size, this observation should not be interpreted as evidence against the slight male predominance consistently reported in larger epidemiological studies [2].

Comparable observations regarding age distribution were reported in the Fez series, a retrospective descriptive study of 23 patients treated in the pediatric oncology and radiology departments of Hassan II University Hospital between January 2015 and January 2020. In that cohort, the median age at diagnosis was six years (range: 15 months to 14 years), and the highest incidence was observed in the two- to five-year age group, accounting for 43.5%. In contrast to our results, a male predominance with a sex ratio of 1:3 was demonstrated, with boys accounting for 57% of cases [15].

Regarding anatomical distribution, the head and neck (non-parameningeal) region was the most common site in our series, representing 25% of cases, followed by the orbit, extremities, and bladder (16.7% each). The genitourinary tract (excluding the bladder and prostate), abdomen, and trunk each accounted for 8.3% of cases. These findings are broadly consistent with those reported by the Intergroup Rhabdomyosarcoma Study Group (IRSG) and the COG, which identified the head and neck region as the most frequent primary site, comprising 27.6% of cases. Nevertheless, variations were noted in the distribution of the remaining anatomical locations, particularly within the genitourinary tract and extremities [16]. Likewise, the series from Fez reported the head and neck region as the most frequent tumor location, accounting for 47.8%, followed by the genitourinary tract at 26.1%. The remaining 26.1% of tumors arose from other sites [15].

As for histological type, the predominance of embryonal RMS observed in our cohort aligns with the literature. The IRS III-IV study reported that 67% of RMS cases were embryonal, 25% alveolar, and 8% pleomorphic. [2] Similarly, data from the Surveillance, Epidemiology, and End Results (SEER) program revealed that embryonal RMS accounted for 57% of pediatric cases, followed by alveolar RMS (23%). By comparison, the other less common types accounted for 1.5% for pleomorphic RMS and 0.6% for sclerosing RMS [17]. In our study, the embryonal RMS represented 66.6% of cases, while alveolar RMS and the botryoid variant each accounted for 16.7%. The embryonal subtype was mainly located in the head and neck region. The alveolar subtype was observed only in the extremities and trunk. Given the limited number of cases, this finding should be interpreted as a cohort-specific observation rather than a definitive site-specific distribution, whereas the botryoid variant was seen in the bladder and genitourinary tract.

Immunohistochemistry continues to play a central role in the diagnosis of RMS. A large study conducted by the COG reported statistical analysis of the diagnostic performance of the four myogenesis-related immunohistochemical markers; myogenin and MyoD1 each achieved a sensitivity of 97%, with specificities of 90% and 91%, respectively. Desmin demonstrated the highest sensitivity (99%) but a lower specificity (71%), while actin exhibited a sensitivity of 94% [18]. In the present series, all cases demonstrated diffuse positivity for desmin and myogenin. Although molecular testing was not routinely available, the immunohistochemical findings were consistent with the diagnosis of rhabdomyosarcoma and supported histological classification.

Several limitations of this study should be acknowledged. First, the retrospective nature of the study design may have introduced selection and information biases due to reliance on medical records that were potentially incomplete or contained missing data. Second, the study was conducted at a single institution and included only 12 patients, which limited the statistical power and generalizability of our findings to a wider pediatric population. Third, the small sample size precluded meaningful subgroup analyses of prognostic factors and survival outcomes. Fourth, molecular and genetic testing was not routinely available, preventing the assessment of important molecular markers such as PAX3-FOXO1 and PAX7-FOXO1 fusion status, which are increasingly important for diagnosis, risk stratification, and prognosis. Fifth, representative gross pathological photographs were not routinely archived and were therefore unavailable for inclusion in the manuscript. Finally, incomplete follow-up data for some patients restricted our ability to comprehensively evaluate long-term outcomes and treatment-related sequelae. Nevertheless, despite these inherent limitations, our study offers valuable epidemiological, clinicopathological, and therapeutic insights into pediatric RMS in Eastern Morocco, a region where such data remain scarce.

## Conclusions

RMS remains the most common soft tissue sarcoma in children and is a heterogeneous malignancy with diverse clinical, histological, and prognostic characteristics. In this retrospective study of 12 pediatric cases diagnosed at Mohammed VI University Hospital Center in Oujda, the disease predominantly affected young children, with a median age of 2.5 years, and showed an equal distribution between males and females. The head and neck region was the most frequent anatomical site, while embryonal RMS was the predominant histological subtype, consistent with findings reported in the literature. Immunohistochemical analysis demonstrated diffuse positivity for desmin and myogenin in all tested cases, highlighting the essential role of immunohistochemistry in establishing the diagnosis and supporting histological classification. A substantial proportion of patients presented with metastatic disease at diagnosis, emphasizing the aggressive nature of the disease and the importance of early detection. Treatment relied on a multidisciplinary approach combining chemotherapy, surgery, and radiotherapy according to individual clinical characteristics and risk stratification. Despite therapeutic advances, mortality remained considerable within our cohort. Although limited by its retrospective design and small sample size, this study provides valuable epidemiological, pathological, and clinical data on pediatric RMS in Eastern Morocco. Larger multicenter studies integrating molecular and genetic analyses are needed to further characterize the disease and improve risk stratification, therapeutic management, and patient outcomes.

## References

[1] Wexler LH, Meyer WH, Helman LJ (2011). Rhabdomyosarcoma and the undifferentiated sarcomas. Principles and Practice of Pediatric Oncology.

[2] Boué DR, Parham DM, Webber B, Crist WM, Qualman SJ (2000). Clinicopathologic study of ectomesenchymomas from Intergroup Rhabdomyosarcoma Study Groups III and IV. Pediatr Dev Pathol.

[3] Gurney JG, Young JL Jr, Roffers SD (1999). Soft tissue sarcomas. Cancer Incidence and Survival Among Children and Adolescents: United States SEER Program 1975-1995.

[4] Leonard H. Wexler (2026). Wexler LH: a periodical for the sarcoma community. Sarcoma Help News.

[5] Ruymann FB, Maddux HR, Ragab A (1988). Congenital anomalies associated with rhabdomyosarcoma: an autopsy study of 115 cases. A report from the Intergroup Rhabdomyosarcoma Study Committee (representing the Children's Cancer Study Group, the Pediatric Oncology Group, the United Kingdom Children's Cancer Study Group, and the Pediatric Intergroup Statistical Center). Med Pediatr Oncol.

[6] 6] D’Andon. A, Hartmann. O, Vassal. G. https://www.gustaveroussy.fr/node/2266 (2025). Malignant mesenchymal tumors or soft-tissue sarcomas (website in French). may.

[7] Dziuba I, Kurzawa P, Dopierała M, Larque AB, Januszkiewicz-Lewandowska D (2018). Rhabdomyosarcoma in children - current pathologic and molecular classification. Pol J Pathol.

[8] (2013). WHO Classification of Tumours of Soft Tissue and Bone. http://publications.iarc.fr/Book-And-Report-Series/Who-Classification-Of-Tumours/WHO-Classification-Of-Tumours-Of-Soft-Tissue-And-Bone-2013.

[9] Agaram NP (2022). Evolving classification of rhabdomyosarcoma. Histopathology.

[10] Sorensen PH, Lynch JC, Qualman SJ (2002). PAX3-FKHR and PAX7-FKHR gene fusions are prognostic indicators in alveolar rhabdomyosarcoma: a report from the children's oncology group. J Clin Oncol.

[11] Dias P, Chen B, Dilday B (2000). Strong immunostaining for myogenin in rhabdomyosarcoma is significantly associated with tumors of the alveolar subclass. Am J Pathol.

[12] Rekhi B, Upadhyay P, Ramteke MP, Dutt A (2016). MYOD1 (L122R) mutations are associated with spindle cell and sclerosing rhabdomyosarcomas with aggressive clinical outcomes. Mod Pathol.

[13] Li G, Ogose A, Kawashima H (2009). Cytogenetic and real-time quantitative reverse-transcriptase polymerase chain reaction analyses in pleomorphic rhabdomyosarcoma. Cancer Genet Cytogenet.

[14] Watson S, Perrin V, Guillemot D (2018). Transcriptomic definition of molecular subgroups of small round cell sarcomas. J Pathol.

[15] Aouatif H (2021). Rhabdomyosarcoma in Children in All Locations: Diagnosis, Radiological Findings, Therapeutic Management, and a Literature Review (Thesis No. 164/21). https://toubkal.imist.ma/handle/123456789/24823.

[16] Qualman S, Lynch J, Bridge J, Parham D, Teot L, Meyer W, Pappo A (2008). Prevalence and clinical impact of anaplasia in childhood rhabdomyosarcoma : a report from the Soft Tissue Sarcoma Committee of the Children's Oncology Group. Cancer.

[17] Ognjanovic S, Linabery AM, Charbonneau B, Ross JA (2009). Trends in childhood rhabdomyosarcoma incidence and survival in the United States, 1975-2005. Cancer.

[18] Morotti RA, Nicol KK, Parham DM (2006). An immunohistochemical algorithm to facilitate diagnosis and subtyping of rhabdomyosarcoma: the Children's Oncology Group experience. Am J Surg Pathol.

